# P-996. Revamping Antimicrobial Stewardship at a Community Safety-Net Hospital: A Tiered, Physician- and Pharmacy-Led model

**DOI:** 10.1093/ofid/ofaf695.1195

**Published:** 2026-01-11

**Authors:** Maria Rosa Velasquez, Alfonso Llosa, Hali Ho, Jiachen Xu, Yam-Yuen Lam, Le Tu Ho, Chike Igboechi, Kuldeep Ghosh, John T Pellicone

**Affiliations:** New York Medical College - NYCHHC/Metropolitan, New York, New York; New York Medical College - NYCHHC/Metropolitan, New York, New York; Metropolitan Hospital, New York, New York; Metropolitan Hospital, New York, New York; Metropolitan Hospital, New York, New York; Metropolitan Hospital, New York, New York; Metropolitan Hospital, New York, New York; New York Medical College - NYCHHC/Metropolitan, New York, New York; Metropolitan Hospital, New York, New York

## Abstract

**Background:**

As antimicrobial resistance escalates globally, stewardship programs have become essential to safeguard antibiotic effectiveness and promote evidence-based care. In October 2022, our institution—a community hospital within New York City Health + Hospitals (NYCHHC)—implemented a restructured, physician- and pharmacist-led antimicrobial stewardship program (ASP) that employs a tiered antibiotic restriction framework, prospective case reviews, structured escalation pathways, and monthly deviation reporting. Policies and tier structures are reviewed annually based on updated antibiograms and feedback (Figure 1). This study assesses the ASP’s impact on local susceptibility patterns and explores its strengths and challenges.

**Methods:**

Retrospective analysis of cumulative yearly antibiograms (2022–2024) from 7 NYCHHC facilities. Hospitals 1–3 are community-based, and 4–7 provide tertiary care. Local ASP compliance was measured as the proportion of accepted interventions among total interventions issued. Intra- and inter-institutional comparisons of pathogen-specific susceptibility rates were conducted using chi-square or Fisher’s exact tests, with statistical significance at p < 0.05.

**Results:**

ASP intervention compliance improved from 82% (300/366) in 2022 to 89.3% (260/291) in 2023 and 92.1% (338/367) in 2024. In our institution (Hospital 1), a positive trend in antimicrobial susceptibility was noted for *Enterococcus faecium, Escherichia coli*, and *Pseudomonas aeruginosa. E. faecium* local inpatient vancomycin susceptibility increased from 28% in 2023 to 75% in 2024, reaching statistical significance (p = 0.032). We noted similar trends among other organisms not included in the primary analysis due to missing data or clinical relevance. Inpatient susceptibility patterns generally improved, while ICU trends showed greater variability (Figures 2–4).

**Conclusion:**

Implementing a tailored ASP model in a community hospital was associated with improved stewardship compliance and trends in susceptibility. Annual tier updates, pathogen- and ICU-specific surveillance, and non-punitive educational interventions were critical drivers. Sustained, targeted efforts, particularly in ICU stewardship and care transitions, will be key to long-term success.

**Disclosures:**

All Authors: No reported disclosures

